# Rheology and Moisture-Responsive Adhesion of Hydroxypropyl Cellulose-Enhanced Polyvinylpyrrolidone–Polyethylene Glycol Hydrogels

**DOI:** 10.3390/gels11120974

**Published:** 2025-12-03

**Authors:** Anna Borisovna Karabanova, Sergey Olegovich Ilyin, Anna Vladimirovna Vlasova, Sergey Vyacheslavovich Antonov

**Affiliations:** A.V. Topchiev Institute of Petrochemical Synthesis, Russian Academy of Sciences, 29 Leninsky Prospect, 119991 Moscow, Russia; karabanova@ips.ac.ru (A.B.K.);

**Keywords:** hydrogel adhesives, polyvinylpyrrolidone, hydroxypropyl cellulose, water content, adhesion, rheology

## Abstract

Despite extensive use of polyvinylpyrrolidone (PVP)–polyethylene glycol (PEG) hydrogels in biomedical adhesives, a systematic understanding of how water content governs their rheological and adhesive performance remains lacking—particularly under variable humidity. This work addresses this gap by introducing 3–12 wt% hydroxypropyl cellulose (HPC) as a non-covalent crosslinker into a PVP/PEG gel (2/1 wt/wt) to tune its moisture uptake and stabilize viscoelasticity, thereby enabling robust, humidity-adaptive adhesion. Analysis of water content in hydrogels across a relative humidity range of 3% to 100% revealed that HPC restricts their water absorption capacity, thereby enhancing their tolerance to high-humidity conditions. The adhesive and rheological properties of the hydrogels were investigated as functions of HPC and water concentrations. With an increase in the HPC content, the adhesive properties of the initial low-water hydrogels decreased. However, high humidity strongly affected the hydrogels’ adhesive and rheological properties. The water content for hydrogels to maintain their adhesive properties was about 7–16%, depending on the hydrogel composition. This range corresponds to relative air humidity of 45–80%, tending to shift towards more moisture conditions under the effect of HPC. Thus, HPC enables PVP/PEG adhesives to operate over a broader range of relative humidities and in contact with wet skin when used in medicine as matrices for transdermal therapeutic systems, wound dressings, and flexible electrodes.

## 1. Introduction

Hydrogels are a special class of materials based on polymers that are hydrophilic in nature [1,2,3]. They form a three-dimensional network structure that consists of polymer chains, bonded either covalently or non-covalently, and this structure can absorb a large amount of water, many times exceeding the dry weight of the polymers [4]. In most cases, hydrogels are multicomponent systems [5], and one of the advantages of these materials is that their properties can be tailored by changing the composition and concentration of components, preparation conditions, or external factors [6]. These features provide hydrogels such properties as high elasticity [7,8], similarity in mechanical characteristics to human tissues [9,10], self-healing [11,12], antibacterial activity [13,14], electrical conductivity [15,16], and other desirable properties [17,18]. Most hydrogels also have good biocompatibility, which makes them promising candidates for use in biomedicine [18,19,20,21], including immunotherapy [22,23], drug delivery [24,25], and tissue engineering [26,27].

Many medical devices based on hydrogels, such as electrodes [28,29], photonic devices [30], sensors [31,32,33,34], wound dressing [35,36,37], and patches [38,39,40], need to be adhesive. Pressure-sensitive adhesives (PSAs) are widely used in industry [41], biomedical engineering [42], and medicine [43]. These viscoelastic polymer systems form strong adhesive bonds with substrates of various chemical natures under a light external pressure applied for a short time [44]. There are many different hydrogel-based PSAs [45]. The strength of their adhesive bonding usually depends on a combination of diffusion, viscoelasticity, and relaxation properties [46]. However, the quality of the PSA contact with a surface of interest also determines its adhesion and many functional properties of the final PSA-based products, for example, the intimate contact between a medical device and a patient’s skin or mucosa, as any voids between a substrate and a sensor would distort signals and reduce the device’s efficiency [47]. In addition, the development of transdermal therapeutic patches activated by electric voltage, light, or other stimuli essentially demands ensuring good adhesion of the patch to the skin for effective drug delivery to the patient’s body [48,49,50].

In the development of bioadhesive systems, the viscoelastic properties of materials require careful consideration, as rheological analysis enables quantitative assessment of stiffness and elasticity—the key parameters governing the adhesive performance of polymer materials [51,52,53]. Thus, the rheology of hydrogels has great practical importance because it controls their adhesive properties, although the relationship between rheology and adhesion in pressure-sensitive adhesives is still under research. Many factors influence the rheology of hydrogels, including the nature and topology of the bonds within them. The topology can depend on the type of bonds—covalent and hydrogen bonds, van der Waals interactions, and noncovalent complexes that may form because of the presence of specific functional groups and water within the polymer network [54].

Water plays a crucial role in the manifestation of hydrogel properties. It maintains the elasticity and dimensional stability of hydrogels by filling voids between polymer chains and facilitates the diffusion of substances within the material, which is essential for biomedical applications and drug delivery [55,56]. Hydrophilic properties of hydrogels are often determined by the presence of specific chemical groups in the polymer backbone chains forming their networks: –OH, –COOH, –COO–, >C=O, >CHNH_2_, −CONH_2_, and –SO_3_H [57,58]. Moreover, water content in hydrogels significantly affects their adhesion characteristics. As a rule, high water concentrations prevent most hydrogels from achieving strong adhesion to a substrate, as water molecules on the contact surface form a boundary layer that weakens interfacial adhesion [59]. Therefore, an essential task for developing biomedical devices based on hydrogels is to consider this aspect and understand how water content and other factors affect adhesion between hydrogel adhesives and substrates. Water also affects the rheological properties of hydrogels. It has a strong plasticizing effect on hydrophilic polymers, decreasing their glass transition temperature. Polymer plasticization by water increases intermacromolecular distances, enhances segmental mobility of main chains, and reduces viscosity, improving flexibility and extensibility of polymers [60]. However, an excessive amount of moisture in a hydrogel may cause deterioration in its functional properties, such as cohesion strength and adhesion.

Although many studies have been conducted on hydrogel materials, there are no papers that comprehensively analyze the dependence of hydrogels’ adhesive and viscoelastic characteristics on water content, which is important for their practical applications, such as wound dressing, transdermal patches, electrodes for electroencephalography, and so on. The article by Tordi et al. [61] investigates multiresponsive ionic conductive alginate/gelatin organohydrogels, focusing on how water content influences their mechanical and conductive properties for wearable electronics. The study highlights the synergy between polymer networks and ion conductivity modulated by hydration level. Gun’ko et al. [57] examined the types of water bound in hydrogels and the effect of varying water content on molecular interactions and polymer network structure. Their work primarily addressed water freezing behavior and microstructural changes, without direct emphasis on mechanical adhesion or rheology. Nishida et al. [62] studied hydrogels with movable cross-links, demonstrating how water content affects mechanical toughness and self-healing behavior through reversible hydrophobic interactions. While this research highlighted mechanical relaxation, it did not jointly assess viscoelasticity and adhesion. Yue et al. [63] designed ultra-high-water-content hydrogels that combine chemical crosslinking and physical entanglement to optimize toughness and crack resistance. Their analysis centered on water-content-influenced mechanical robustness and structural stability, without a combined focus on adhesion or rheological properties.

In this context, this work aims to investigate the effect of water content on the properties of hydrogel adhesives based on non-covalently bonded complexes. Systems based on polyvinylpyrrolidone (PVP) and polyethylene glycol (PEG) were chosen for further research. PVP is a well-known hydrophilic polymer with good biocompatibility and biodegradation profile, which is widely used in medicine and cosmetology [64,65,66]. PEG was utilized as a plasticizer in the hydrogel composition. It is known that PVP and PEG-400 form a unique interpolymer complex in combination with water, exhibiting outstanding adhesive properties at a specific ratio between components [67]. Hydroxypropyl cellulose (HPC) was used to modify the PVP/PEG hydrogel system. HPC is often introduced into a polymer matrix in conjunction with PVP for manufacturing medical products, as they are miscible in water, while their ratio controls the drug release rate [68,69,70]. In our case, HPC was expected to work as a non-covalent crosslinking agent to create a stronger hydrogel adhesive with higher moisture resistance.

## 2. Results and Discussion

### 2.1. Characterization of PVP/PEG Hydrogels Modified with HPC

#### 2.1.1. IR Analysis of PVP/PEG Hydrogels Modified with HPC

**Figure 1:**
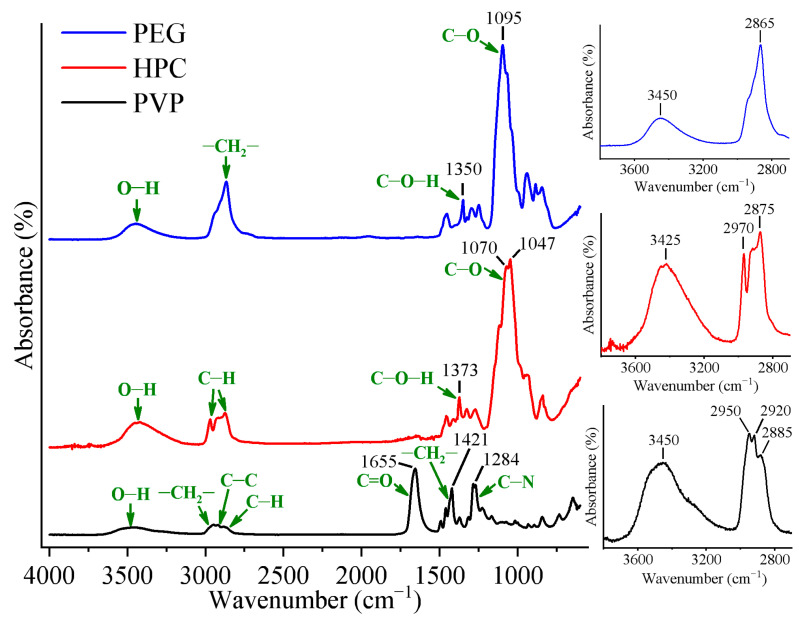
IR spectra of pure polyvinylpyrrolidone (PVP), polyethylene glycol (PEG), and hydroxypropyl cellulose (HPC). The insets show absorption bands due to O–H and C–H stretching vibrations.

Figure 1 demonstrates IR spectra of pure hydrogel components—PVP, PEG, and HPC. Broad bands were observed in the range from 3100 cm^−1^ to 3650 cm^−1^ in the IR spectra for all samples. Since the samples were pre-dried in an oven, the presence of free water was minimized. Vibrations of free O-H groups usually correspond to wavenumbers above 3500 cm^−1^ (e.g., 3755 cm^−1^ [64]), while the bands on the obtained spectra are located in the range of 3400–3500 cm^−1^, which indicates the stretching of hydroxyl groups in the intermolecular and intramolecular hydrogen bonding [71]. Given the fact that there is no hydroxyl group in the molecular structure of PVP, the band around 3450 cm^−1^ on the PVP spectra can result from the absorbed water that forms hydrogen bonds with PVP molecules. Other characteristic bands for PVP are 2950 cm^−1^ (–CH_2_– stretching), 2920 cm^−1^ (C=O stretching), 2885 cm^−1^ (C–H stretching), 1655 cm^−1^ (C=O stretching), 1421 cm^−1^ (–CH_2_– bending), and 1284 cm^−1^ (C–N stretching) [72]. In the case of PEG, a wide band is located in the region of 3450 cm^−1^ because of the stretching of hydroxyl groups (O-H) in the PEG structure. The intense band around 1095 cm^−1^ is associated with C-O stretching vibrations, while the second most intense band close to 2865 cm^−1^ corresponds to –CH_2_– stretching vibrations. In addition, (C–O–H) deformation band is observed in the range of 1300–1400 cm^−1^ [73]. In the case of HPC spectra, the O-H stretching band (*ν* = 3425 cm^−1^) is located at lower wavenumbers in comparison with PVP and PEG spectra, which may result from stronger hydrogen bonding. The bands of stretching of methyl groups (–CH_3_) and hydroxypropyl groups (–C_3_H_5_O) are located at 2800–3000 cm^−1^ [74]. The band at 1373 cm^−1^ characterizes the C–O stretching vibration in pyranose cycle, while the band located at 1040–1070 cm^−1^ is indicative of the stretching vibration of the ethereal C–O–C bond.

**Figure 2:**
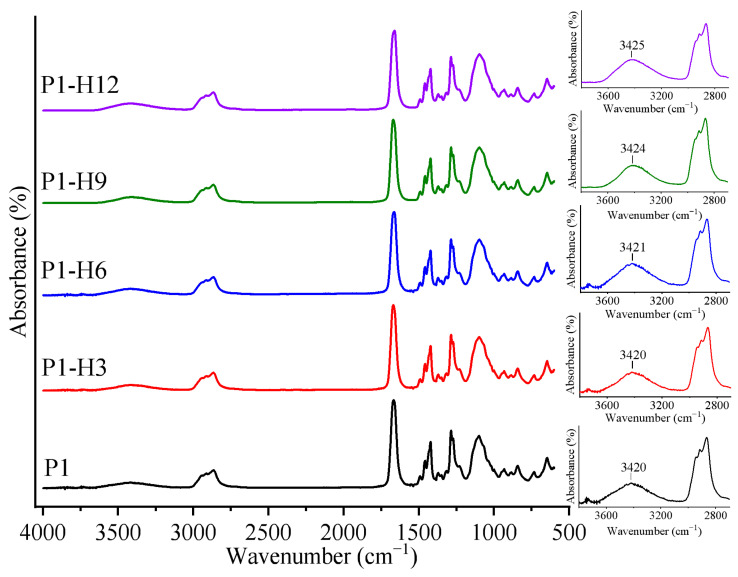
IR spectra of PVP/PEG hydrogels (2/1 wt/wt) modified with different HPC contents: 0 (P1), 3 (P1-H3), 6 (P1-H6), 9 (P1-H9), or 12 wt% (P1-H12). The insets show absorption bands due to O–H and C–H stretching vibrations.

In the case of the PVP/PEG and PVP/PEG/HPC hydrogels (Figure 2), there are no new absorption bands that are absent in the individual polymers, indicating the lack of chemical interactions. Nevertheless, strong interactions between PVP, PEG, and HPC, and the resultant structuring of their mixtures, may be caused by the formation of hydrogen bonds between different functional groups of the components. The HPC structure features both H-donors (hydroxyl groups) and H-acceptors (oxygen atoms in pyranose rings) [75]. PVP contains only acceptor H-bonding carbonyl oxygen in the ring structure. In contrast, short-chain PEG-400 carries two proton-donating terminal hydroxyl groups [76]. In this manner, hydrogen bonds can form between PVP carbonyl groups and HPC hydroxyl groups [70], as well as between PEG hydroxyl groups and the oxygen in the pyranose ring of HPC. The interactions of PVP and PEG can occur through the formation of H-bonds between the proton-donor hydroxyl groups of PEG molecules with the PVP electron-donating carbonyl oxygen atoms [45]. In addition, HPC can form intramolecular H-bonds [77]: most often, between different hydroxyl groups or between a hydroxyl group and a pyranose oxygen of HPC [78]. Moreover, residual or absorbed water can also form H-bonds with other hydrogel components.

In our case, the O–H vibration band for the PVP/PEG hydrogel shifted towards a lower wavenumber equal to 3420 cm^−1^, confirming the formation of the PVP–PEG complex due to hydrogen bonding, which is stronger than interaction with absorbed water in the initial two polymers (Figure 2). Upon addition of HPC to the hydrogel formulation, the O–H stretching band shifts to higher wavenumbers, reaching 3425 cm^−1^ for the sample containing 12 wt% HPC. This fact indicates the contribution of HPC in the formation of intra- and intermolecular hydrogen bonds, including their weakening in comparison with the original PVP/PEG hydrogel.

#### 2.1.2. Swelling and Water Content in PVP/PEG Hydrogels Modified with HPC

**Figure 3:**
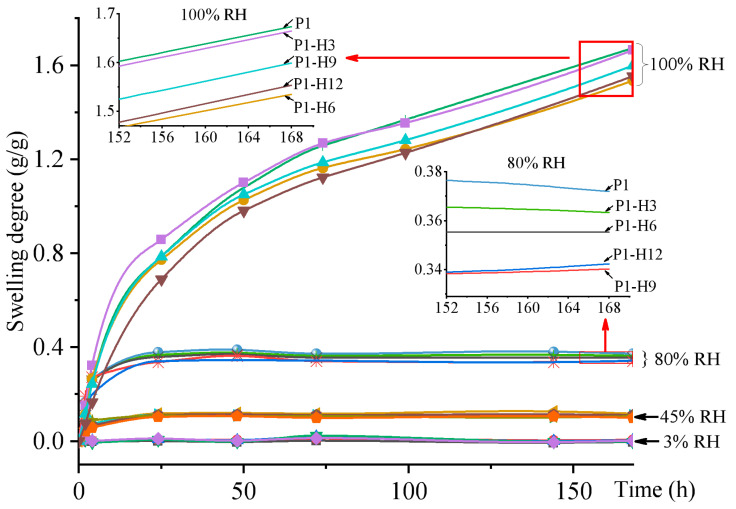
7-Day swelling curves of PVP/PEG and PVP/PEG/HPC hydrogels at different relative humidities (RH). The insets show enlarged areas of the curves.

The process of hydrogel swelling at different RH as a function of time is shown in Figure 3. As can be seen from the graph, the swelling degree for all samples at 100% RH was increasing steadily throughout all 7 days, not achieving the equilibrium water content. It is worth noting that all hydrogels at 100% RH during their long-term conditioning formed a texture similar to a viscous liquid, which was inappropriate for practical use as hydrogel materials. With the decrease in humidity, the overall picture has changed. On the 2nd day of exposure in desiccators with controlled humidity, the weight of hydrogel samples reached a plateau at an RH equal to 3%, 45%, or 80%. Minor fluctuations in swelling degree within ±0.01 g/g can be associated with the opening of the desiccators at the time of weighing the samples. The addition of HPC decreases the ability of the PVP/PEG hydrogel to absorb water and swell, but to a limited extent. For example, 3% and 9% HPC reduce the swelling degree by 3% and 9%, respectively, at 80% RH. With the decrease in relative humidity, the range of swelling degree values was narrowing. As a consequence, the swelling curves at 3% and 45% RH for all formulations with various HPC content were almost identical.

**Figure 4:**
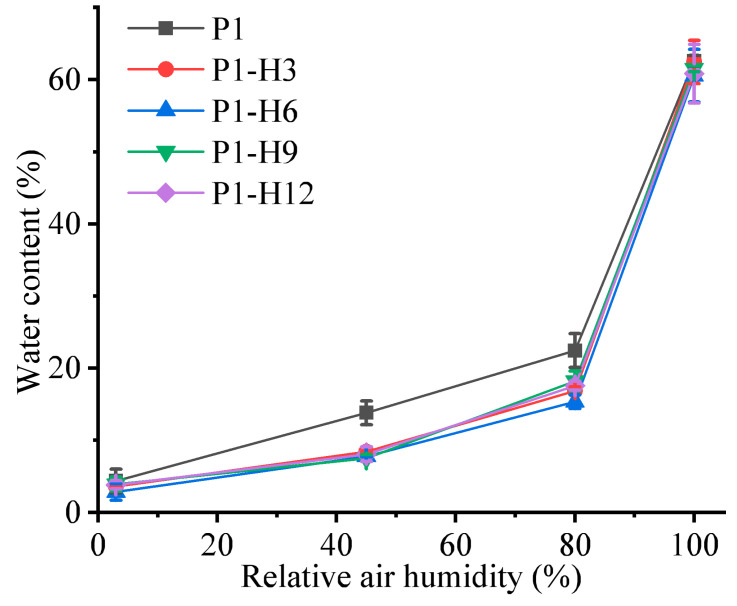
Isotherms of water vapor sorption by PVP/PEG hydrogels modified with different HPC concentrations. The values at 100% RH are non-equilibrium ones.

The absorption of water by a polymer in equilibrium with the relative humidity is depicted by its moisture-sorption isotherm [79]. The results of our measurements of the water content in the hydrogels by Karl Fischer titration are shown in Figure 4. As was demonstrated above, the experimental points measured at 100% RH cannot be regarded as equilibrium ones. To evaluate and describe the absorption of water vapor by hydrophilic polymers the Brunauer–Emmett–Teller (BET) theory is widely used [80,81,82]. According to the classification by this theory, the resulting isotherms for the PVP/PEG-based hydrogels have type II sigmoidal shapes [83]. The type II isotherm corresponds to nonporous or microporous hydrophilic materials [84], such as the hydrogels under consideration.

At about 3–4% RH, the equilibrium water content in both the initial PVP/PEG gel and the hydrogels containing HPC was almost the same: 2.5–4.5% of their weight (Figure 4). With an increase in the relative humidity, the water content in the HPC-containing hydrogels became lower in comparison with the corresponding HPC-free hydrogels. At 45% RH, the water content in the initial PVP/PEG hydrogel was 13–14%, while it declined to 7.8–8.4% for the hydrogels modified with HPC. At the same time, the increase in the HPC concentration did not lead to significant changes in the water content. A similar pattern was observed with further growth in the relative humidity up to 80%. The addition of HPC limited the ability of the PVP/PEG hydrogel to swell, as the equilibrium water content decreased from 22.5% for the initial hydrogel to 15–18% for the modified samples. When the relative humidity was adjusted to 100%, all samples showed approximately the same water contents of 60–62% after 7 days in the desiccator, although these values are non-equilibrium. Thus, the addition of HPC reduces the equilibrium water content in the hydrogels, which may result from the structural limitations imposed by denser non-covalent crosslinking provided by HPC. This modest but systematic reduction in equilibrium water uptake (e.g., from 22.5% to 15–18% at 80% RH) contrasts with organohydrogel systems such as alginate/gelatin/glycerol, where water content remains nearly constant (≈14%) across 20–80% RH due to strong glycerol–water hydrogen bonding [61]. Notably, enzymatically crosslinked, fully bio-based gelatin organohydrogels exhibit comparable humidity stability under cyclic RH exposure—a key design goal for long-term wearable sensors [85]. In our aqueous PVP/PEG/HPC system, HPC does not fully exclude water (as glycerol does), but modulates its plasticizing effect—enabling water content to remain in the 7–16% “adhesion window” over a higher RH range (45–80% versus 20–60% in Mn^2+^-crosslinked alginate/gelatin gels [61]. This confirms that HPC acts not as a moisture barrier, but as a buffer that delays over-plasticization while preserving the reversible, stimuli-responsive character of hydrogel adhesives.

In addition, HPC strongly interacts with PVP and PEG molecules via H-bonds, thereby potentially excluding their H-donor/H-acceptor sites from interacting with water molecules. At least, HPC makes the hydrogels less hydrophilic, as its presence shifts the O–H stretching band of IR spectra to higher wavenumbers (see the insets in Figure 2), indicating a weakening of hydrogen-bonding interactions.

### 2.2. Adhesion Properties of PVP/PEG Hydrogels Modified with HPC

#### 2.2.1. Tack Testing

**Figure 5:**
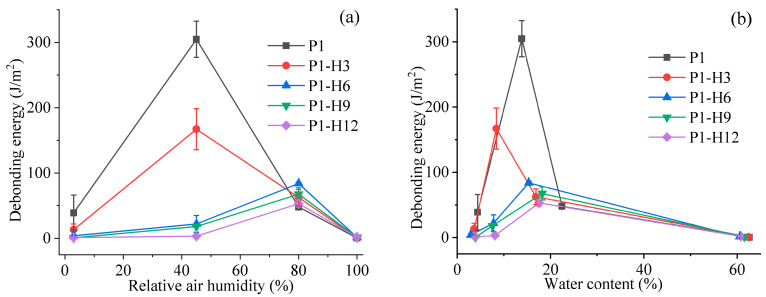
Debonding energy dependence on relative humidity (**a**) or water content (**b**) for PVP/PEG hydrogels modified with HPC.

**Figure 6:**
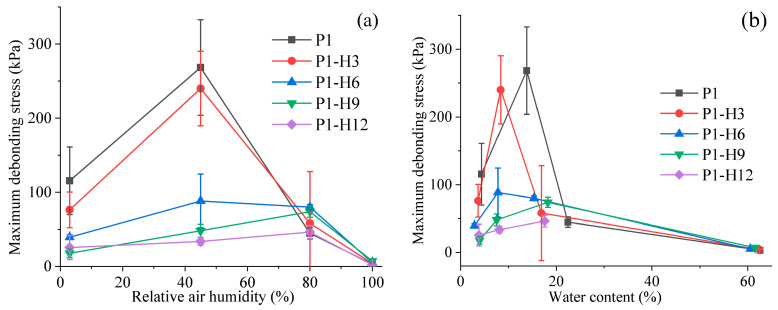
Maximum debonding stress dependence on relative humidity (**a**) or water content (**b**) for PVP/PEG hydrogels modified with HPC.

The probe tack test was performed on the hydrogel samples kept in four desiccators with different relative humidities (3%, 45%, 80%, or 100%). The results are shown in Figure 5 and Figure 6. At 3% RH, all hydrogel samples were too rigid to demonstrate good tackiness or adhesive strength. As the relative humidity rose to 45%, the tackiness improved significantly. In these cases, the detachment of the steel rod from all hydrogel surfaces proceeded with an adhesive-type fracture. The debonding energy and maximum debonding stress decreased proportionally with an increase in the HPC content in the hydrogels.

A further increase in relative humidity to 80% resulted in a significant reduction in the adhesive parameters for both the initial PVP/PEG hydrogel (P1) and the hydrogel containing 3% HPC (P1-H3) due to deterioration in cohesive properties. However, for the hydrogels with 6%, 9%, and 12% HPC, these parameters became higher. For all samples, the failure mode shifted to cohesive fracture, indicating a reduction in cohesive strength with increasing water content. In other words, the destruction of the adhesive joint is occurring within the hydrogel volume rather than at the interface with the substrate. When the relative humidity increased to 100%, all samples practically lost their cohesive strength and turned into viscous liquids, indicating that the polymer components of the hydrogels had dissolved completely in the absorbed water.

The magnitude of humidity-induced adhesion loss in the unmodified PVP/PEG hydrogel (e.g., 78% drop in the maximum debonding stress from 45% to 80% RH, Figure 6a) is comparable to the relative humidity sensitivity (relative change of resistance Δ*R*/*R*_0_ per %RH) reported for Mn^2+^-crosslinked alginate/gelatin organohydrogels (0.022%^−1^, [61]), confirming that unmodified hydrophilic networks are intrinsically humidity-labile. Crucially, however, while Tordi et al. achieve RH tolerance by suppressing water uptake via glycerol plasticization and cationic crosslinking [61,86], we retain hydration-driven tack but shift its operational window—demonstrating a distinct design philosophy: adaptive adhesion, not humidity immunity.

#### 2.2.2. Peel Testing

**Figure 7:**
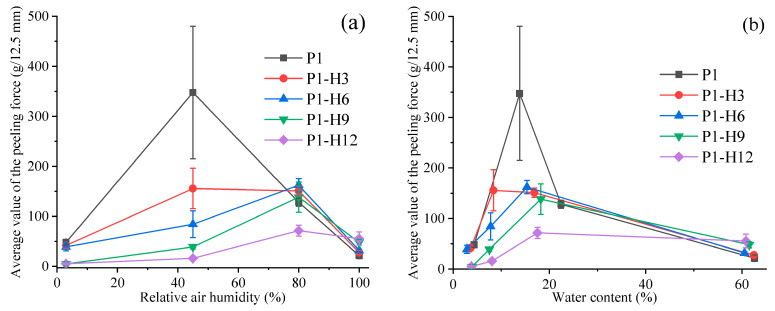
Dependence of the average peeling force on relative humidity (**a**) or water content (**b**) for PVP/PEG hydrogels modified with HPC.

Figure 7 shows the peel force of hydrogel adhesives at a peel angle of 90° as a function of relative humidity. As in the case of probe tack tests, similar dependences of adhesive properties were observed with changes in relative humidity and HPC concentration. At 3% RH, the samples were almost non-adhesive and showed very low peel force values. At 45% RH, the peel force increased significantly for all hydrogels, but the addition of HPC caused a gradual decrease in the peel force in comparison with the initial HPC-free hydrogel. For all samples, the type of failure was adhesive. An elevation of RH to 80% decreased the peel forces for samples P1 and P1-H3 but increased for samples P1-H6, P1-H9, and P1-H12. The failure type changed from adhesive to cohesive. At 100% RH, the samples also demonstrated cohesive breakage but at lower peel forces.

**Figure 8:**
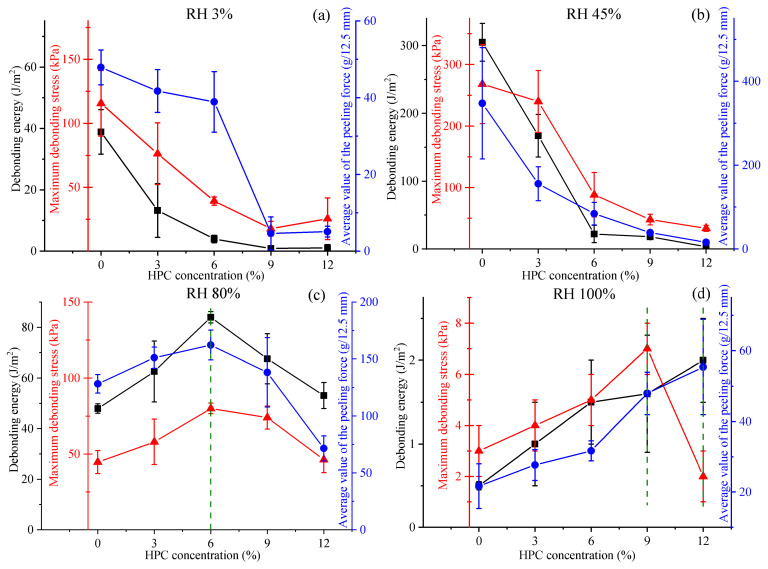
Dependence of debonding energy, maximum debonding stress, and average value of the peeling force on HPC content at a relative humidity of 3% (**a**), 45% (**b**), 80% (**c**), and 100% (**d**).

Thus, water plays a crucial role in the adhesive performance of PVP/PEG-based hydrogels. If there is insufficient moisture in the hydrogel, it becomes rigid and loses its tackiness. On the other hand, if a hydrogel contains too much water, its internal structure changes, while the cohesive strength decreases significantly. This fact leads to the formation of fibrils at peeling and cohesive failure. Therefore, at relative humidity above 50–60%, the failure mechanism changes from adhesive to cohesive. At the same time, the optimum properties for the initial PVP/PEG hydrogel and samples with low HPC content were observed at 45% RH. As the amount of HPC in the hydrogel increases, its region of optimum adhesion properties shifts to higher RH values (Figure 8). This transformation probably indicates the ability of HPC to cross-link the hydrogel by hydrogen bonds, making it more tolerant to high RH. As follows from Figure 5b, Figure 6b and Figure 7b, the maximum values of the adhesive parameters for all hydrogel samples were observed within a relatively narrow range of moisture content—from 7% to 16%.

### 2.3. Rheological Properties of PVP/PEG Hydrogels Modified with HPC

**Figure 9:**
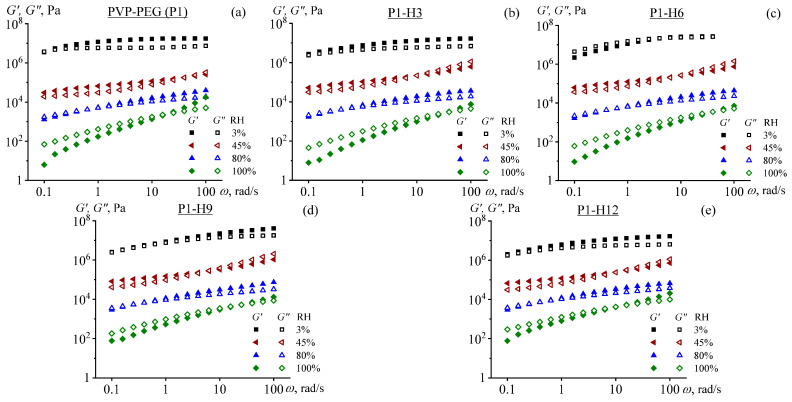
Frequency dependencies of storage and loss moduli for PVP/PEG/HPC hydrogels: P1 (**a**), P1-H3 (**b**), P1-H6 (**c**), P1-H9 (**d**), and P1-H12 (**e**).

Figure 9 shows the frequency dependencies of the storage modulus (*G*′) and loss modulus (*G*″) for the HPC-modified PVP/PEG hydrogels at different relative humidities under which they were stored. The obtained results demonstrate that *G*′ and *G*″ increased steadily with the deformation frequency for all hydrogels. On the contrary, a rise in relative humidity led to a decrease in the values of *G*′ and *G*″. The key factor is the transformation of the relaxation properties of the hydrogels under moisture conditions. At 3% RH and high frequencies, the storage modulus is nearly independent of angular frequency and exceeds the loss modulus, which also shows weak frequency dependence. This region corresponds to the glassy state of the hydrogels, which exhibit a high storage modulus of about 10 MPa and, consequently, do not show noticeable tackiness. Only at low frequencies do the moduli decrease, indicating the onset of the glass-to-rubbery transition.

An increase in relative humidity to 45% converts the hydrogels to a rubbery state, endowing them with adhesion performance. In this relaxation state, the storage modulus is again nearly frequency-independent and exceeds the loss modulus, but its value ranges from about 0.03 to 0.1 MPa, as opposed to the 10 MPa observed in the glassy state. Expectedly, an increase in the angular frequency causes the moduli to rise, and *G*″ becomes greater than *G*′, which is associated with the mechanical glass transition [53]. It means that under these conditions corresponding to rapid impacts, adhesives exhibit higher cohesive strength and, consequently, higher adhesive bond strength, even despite initially having low cohesion.

At 80% RH, the storage and loss moduli decline further, which corresponds to the hydrogels’ transition into an intermediate state between liquid and rubbery [87]. At low frequencies, the loss modulus slightly exceeds the storage modulus, resulting in good wetting ability and tackiness. Conversely, an increase in the frequency elevates the moduli and makes them less frequency-dependent, shifting the hydrogels into the rubbery state, which imparts strength to adhesive bonds during their rapid loading. Finally, at 100% RH, the hydrogels turn into liquids—essentially polymer solutions—for which *G*″ > *G*′ in the low-frequency range corresponding to the terminal zone [53].

The HPC addition does not significantly affect viscoelasticity at RH levels between 3% and 80%, as the frequency dependencies of both the storage and loss moduli remain almost unchanged across the glassy and rubbery state regions. The difference between the curves becomes apparent only at 100% RH, i.e., when transitioning into the liquid-like relaxation regime. In these cases, HPC leads to an increase in both storage and loss moduli at low frequencies, where the weak crosslinking of macromolecules via non-covalent bonds becomes evident [53]. As a result, the HPC-containing hydrogels exhibit significantly higher stiffness (e.g., *G*′ = 9 Pa versus 95 Pa at 0% and 12% HPC, respectively, when *ω* = 0.1 rad/s) and are more prone to storing mechanical energy (*G*′ approaches *G*″), meaning that they better resist cohesive failure under low-speed deformation in high-humidity conditions. This behavior correlates with the results of the adhesion study (Figure 8), where 6% HPC leads to a 75% increase in the apparent value of debonding energy, a 78% increase in the value of maximum debonding stress, and a 27% increase in the apparent value of the peeling force compared with the initial PVP/PEG hydrogel under the high relative humidity of 80%.

The terminal-zone stiffening observed here (*G*′ rising from 9 Pa to 95 Pa at *ω* = 0.1 rad/s, 80% RH, upon increasing HPC from 0% to 12%) reflects physical network reinforcement without compromising compliance. By contrast, in cation-crosslinked alginate/gelatin organohydrogels, the storage modulus lies in the MPa range: Young’s modulus spans 0.52–2.57 MPa depending on the crosslinker (Mn^2+^ to Fe^3+^/Zr^4+^ [61]), corresponding to *G*′ ~ hundreds of kPa to MPa in the rubbery plateau. This difference stems from crosslinking strategy. HPC in PVP/PEG provides reversible, dynamic H-bonding crosslinks that strengthen the network only under high hydration (i.e., in the terminal zone), preserving low-modulus, skin-conforming behavior at intermediate RH (*G*′ ~ 0.03–0.1 MPa at 45% RH). Multivalent cations in alginate/gelatin create permanent, strong ionic crosslinks that rigidify the matrix across all humidity levels, yielding higher baseline stiffness but reduced adaptability to low-pressure, conformal adhesion. Thus, while both systems leverage non-covalent crosslinking for humidity tolerance, our approach prioritizes viscoelastic adaptivity, enabling strong adhesion only when needed (at higher RH), without sacrificing softness under drier conditions.

To classify the type of hydrogel adhesives by their viscoelastic properties, we used the concept proposed by Chang [88]. According to this concept, for most PSAs, the values of *G*′ and *G*″ at room temperature and frequencies of 0.01 s^−1^ and 100 s^−1^ lie within the range from 10^3^ to 10^6^ Pa. Each PSA can be characterized by four values: *G*′ at 0.01 s^−1^ and 100 s^−1^ and *G*″ also at 0.01 s^−1^ and 100 s^−1^. These four characteristic points delineate the so-called viscoelastic window in the *G*′–*G*″ plane. Within this region, the space is divided into four quadrants, each associated with distinct rheological and adhesive behaviors. Briefly, the 1st quadrant includes anti-adhesive coatings, the 2nd quadrant corresponds to adhesives with high resistance to shear loads, the 3rd quadrant represents removable adhesives, and the 4th quadrant signifies adhesives with fast adhesion at low temperatures [89]. General-purpose PSAs that are the most optimal in operational properties are located in the central region of the chart.

**Figure 10:**
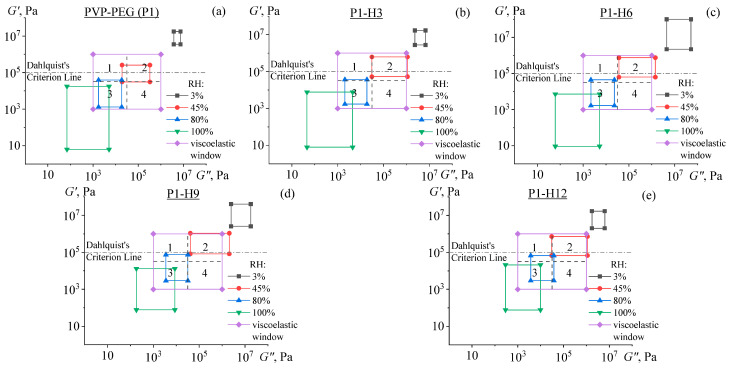
Chang’s viscoelastic windows for PVP/PEG/HPC hydrogels: P1 (**a**), P1-H3 (**b**), P1-H6 (**c**), P1-H9 (**d**), and P1-H12 (**e**).

The viscoelastic windows constructed for the PVP/PEG/HPC hydrogels are shown in Figure 10. The graphs indicate that all windows of hydrogels stored at 45% RH are located in the 2nd quadrant, while the samples kept at 80% RH belong mainly to the 3rd quadrant. At the same time, the samples stored at 3% RH and 100% RH are not PSAs according to Chang’s concept, as they do not fall into the prescribed range of moduli. These results agree well with the conducted studies of hydrogels’ adhesive properties: their films stored in dry air with 3% RH demonstrated very high stiffness and lack of tackiness, whereas the samples kept in the humid condition of 100% RH passed into a liquid state and were not able to form strong adhesive bonds with the substrate. Note that the location of the viscoelastic windows for the hydrogels depends on relative humidity. At 50–60% RH, the hydrogels’ window of viscoelastic properties is located in the central region, corresponding to a general-purpose PSA. This result agrees with previous studies conducted by Feldstein et al., which revealed that the optimum properties of PVP/PEG hydrogels occur at a relative humidity of 45–65% [90]. Thus, the change in relative humidity enables the switching of adhesion characteristics of the PVP/PEG/HPC hydrogels, whereas the addition of HPC shifts the switching point to higher moisture content.

## 3. Conclusions

The investigation of hydrogel adhesives based on a PVP/PEG/HPC mixture revealed that the addition of HPC to the PVP/PEG hydrogel contributed to optimization of its adhesive performance and made it more resistant to high relative humidity. Despite the fact that the addition of HPC had almost no effect on the swelling degree, the adhesive and rheological properties of the hydrogels changed significantly, especially under high moisture conditions. Water alters the relaxation state of the hydrogels, causing their transition from the glassy to the rubbery and then to the liquid state upon an increase in relative humidity of the surrounding atmosphere from 3% to 100%. In turn, HPC acts as a non-covalent crosslinker of the PVP/PEG hydrogels, affecting their viscoelasticity in the terminal zone, i.e., at high humidity and/or long exposure times. High HPC concentrations of 9–12% reduced the adhesion of the hydrogels, but 3–6% HPC provided them with the optimum properties, including the highest apparent adhesive strength at high moisture content. The rheological tests revealed that the hydrogels are in the rubbery state at relative humidities of 45% and 80%, matching the window of viscoelastic properties of typical pressure-sensitive adhesives in agreement with Chang’s concept. At the same time, the hydrogels do not exhibit pressure-sensitive adhesion at 3% or 100% relative humidity, as they are in the glassy or liquid state, respectively. The maximum adhesion characteristics of the PVP/PEG/HPC hydrogels occurred when they contained 7–16% water, corresponding to a relative humidity of 45–80%. In this case, 6% HPC raises the apparent adhesive strength by 78% compared with the initial PVP/PEG hydrogel under a high relative humidity of 80%. The PVP/PEG/HPC hydrogels can be used in various medical applications, including wound dressings, transdermal patches, and flexible electrodes operating at high humidity.

## 4. Materials and Methods

### 4.1. Materials

Polyvinylpyrrolidone Kollidon^®^ K90 (*M*_w_ = 1.2 × 10^6^ g/mol) was purchased from BASF (Ludwigshafen, Germany). Hydroxypropyl cellulose Klucel^™^ LF (*M*_w_ = 9.5 × 10^4^ g/mol, substitution degree ≈ 3) was supplied by Ashland Inc. (Wilmington, DE, USA), while polyethylene glycol (*M*_w_ = 400 g/mol) and ethanol (96%) were purchased from Chimmed (Moscow, Russia).

### 4.2. Methods

#### 4.2.1. Preparation of Hydrogel Films

Preparation of hydrogel samples was carried out by the solvent cast method using PVP and PEG at a weight ratio of 2:1 as a matrix that was filled with 0 wt%, 3 wt%, 6 wt%, 9 wt%, or 12 wt% HPC, named P1, P1-H3, P1-H6, P1-H9, or P1-H12, respectively. The compositions of the samples are presented in Table 1. All components were dissolved in a mixture of distilled water and ethanol taken at a 1:1 weight ratio to form a 20% solution of the respective formulation. The solutions were homogenized using a magnetic stirrer at 25 °C for 4 h. Then, the obtained solutions were left overnight at room temperature until complete dissolution, accompanied by the release of air bubbles that entered the solutions during stirring.

The prepared solutions were poured into silicone molds and dried at room temperature. Silicone molds were covered with paper to prevent rapid drying of the upper layers of the hydrogels and the appearance of bubbles on the resultant film surfaces. The thickness of the dried films was about 400–600 μm. Then, the obtained hydrogel films were conditioned for 7 days in desiccators with different relative humidity (RH) levels—3, 45, 80, or 100%—over H_2_SO_4_ solutions of proper concentrations to achieve different equilibrium water contents in the hydrogel films. The RH values were controlled by a humidity data logger 174H (Testo, Titisee-Neustadt, Germany).

#### 4.2.2. Infrared Spectroscopy

Infrared (IR) spectroscopy was used to identify functional groups and bond types, as well as to evaluate interactions between components in the prepared hydrogels [91]. IR spectra were recorded using an IFS 66 v/s spectrometer (Bruker, Rosenheim, Germany). The measurements were carried out in the range from 600 to 4000 cm^−1^. In all cases, the resolution was 2 cm^−1^. All samples were pre-dried in an oven at 60 °C for 24 h.

#### 4.2.3. Water Content and Swelling Degree Measurements

Before studying the properties of hydrogels, it is necessary to determine the dependence of their actual water content on the RH of the atmosphere where they were stored. Moreover, analysis of the swelling behavior of hydrogels is essential for understanding how their properties change at different RH in practical applications. For these reasons, the dependence of hydrogel swelling degree on the exposure time in air environments with varied humidity levels was studied. The hydrogel samples were pre-dried in an oven at 60 °C for 48 h to remove residual moisture. Then, they were placed in desiccators maintained at 3%, 45%, 80%, and 100% RH and weighed at regular intervals for 7 days—a duration sufficient to reach equilibrium moisture content at 3–80% RH. The swelling degree (*SD*) was calculated using Equation (1):

(1) $$SD=\frac{m_{swollen\;gel}-m_{dried\;gel}}{m_{dried\;gel}},$$

After 7 days, the water content in hydrogels was determined by Karl Fischer volumetric titration on a titrator Expert-007M (Econics-Expert, Moscow, Russia) whose operation principle was based on calculating the water content via the volume of the consumed titrant and Equation (2):

(2) $$\varphi =\frac{V\cdot T}{m}\cdot 100\%,$$

where *V* is the volume of titrant (Fischer’s reagent), *T* is the titer of Fischer’s reagent, and *m* is the mass of the sample under study. AKVA M^®^-Solvent (methanol, imidazole, and SO_2_) and AKVA M^®^-Titrant 2 MN (diethylene glycol monoethyl ether, imidazole, 2-methylimidazole, SO_2_, I_2_, and 1H-imidazole) supplied by Akvametriya (Moscow, Russia) were used as Fischer’s reagents.

#### 4.2.4. Probe Tack Test

The probe tack test was used to simulate contact between the adhesive surface and a steel probe, followed by their detachment. The test allows quantification of the force required to rupture the adhesive joint, as well as identification of the failure mode—adhesive, cohesive, or mixed—including the presence or absence of fibrillation [92]. Tests were performed on a texture analyzer TA.XTplus (Stable Micro System, Godalming, UK). A stainless-steel probe with a flat-ended tip and a diameter of 9.98 mm was used to establish adhesive contact with hydrogel samples at a controlled approach depth of 0.1 mm, a dwell (contact) time of 10 s, and a retraction speed of 0.1 mm/s. The adhesive properties of hydrogel samples were characterized by two parameters: debonding energy (*W*) and maximum apparent debonding stress (*σ*_max_), which were calculated using Equations (3) and (4), respectively:

(3) $$W=\frac{\int_{0}^{h_{max}}Fdh}{S},$$

where *F* is the current pulling force, *h* is the distance traveled by the probe when pulling, *h*_max_ is the distance at the moment of debonding, and *S* is the surface area of the rod’s flat end.

(4) $$\sigma_{max}=\frac{F_{max}}{S},$$

where *F*_max_ is the maximum debonding force.

#### 4.2.5. 90-Degree Peel Test

The 90-degree peel test simulates the process of peeling an adhesive from a substrate, allowing the average value of the peeling force to be estimated. Tests were performed using a TT-1100 tensile tester (Cheminstruments, Fairfield, OH, USA) equipped with a 90° peel fixture to maintain a constant peeling angle throughout the experiment. A hydrogel film (9 cm × 1.25 cm) was bonded to the poly(ethylene terephthalate) foam tape with a thickness of 500 μm (LIKK, Lipetsk, Russia) to avoid its elongation during the experiment. Then, the side of the sample covered with the hydrogel layer was brought in contact with a polished steel plate (12.6 cm × 5.0 cm) and rolled twice with a standard 2 kg roller. The 2 cm piece of the substrate tape was fixed in the grip, and the test was conducted with a linear peeling speed of 3.8 cm/min. The average value of the peeling force was recorded.

#### 4.2.6. Rheological Properties

Measurements of rheological parameters were performed on a rotational rheometer DHR-2 (TA Instruments, New Castle, DE, USA) equipped with a parallel-plate working unit (8 mm diameter, 300–400-μm gap). Components of the complex modulus (storage *G*′ and loss *G*″ moduli) were determined in the region of linear viscoelasticity using a relative strain value of 0.1% and varying the angular frequency from 0.1 to 100 rad/s.

## Figures and Tables

**Table 1:** Formulations of PVP/PEG hydrogels modified with HPC (on a dry basis).

Sample	PVP/PEG at a Ratio of 2:1 (wt%)	HPC (wt%)
P1	100	0
P1-H3	97	3
P1-H6	94	6
P1-H9	91	9
P1-H12	88	12

## Data Availability

The original contributions presented in this study are included in the article. Further inquiries can be directed to the corresponding author.
